# Estimating Childhood Stunting and Overweight Trends in the European Region from Sparse Longitudinal Data

**DOI:** 10.1093/jn/nxac072

**Published:** 2022-03-29

**Authors:** Chitra M Saraswati, Elaine Borghi, João J R da Silva Breda, Monica C Flores-Urrutia, Julianne Williams, Chika Hayashi, Edward A Frongillo, Alexander C McLain

**Affiliations:** Department of Nutrition and Food Safety, WHO, Geneva, Switzerland; Department of Nutrition and Food Safety, WHO, Geneva, Switzerland; Quality of Care Office, WHO Regional Office for Europe, Athens, Greece; Department of Nutrition and Food Safety, WHO, Geneva, Switzerland; Regional Office for Europe, WHO, Geneva, Switzerland; Division of Data, Analytics, Planning and Monitoring, UNICEF, New York, NY, USA; Arnold School of Public Health, University of South Carolina, Columbia, SC, USA; Arnold School of Public Health, University of South Carolina, Columbia, SC, USA

**Keywords:** stunting, overweight, child malnutrition, modeling, data sparsity

## Abstract

**Background:**

Monitoring countries’ progress toward the achievement of their nutrition targets is an important task, but data sparsity makes monitoring trends challenging. Childhood stunting and overweight data in the European region over the last 30 y have had low coverage and frequency, with most data only covering a portion of the complete age interval of 0–59 mo.

**Objectives:**

We implemented a statistical method to extract useful information on child malnutrition trends from sparse longitudinal data for these indicators.

**Methods:**

Heteroscedastic penalized longitudinal mixed models were used to accommodate data sparsity and predict region-wide, country-level trends over time. We leveraged prevalence estimates stratified by sex and partial age intervals (i.e., intervals that do not cover the complete 0–59 mo), which expanded the available data (for stunting: from 84 sources and 428 prevalence estimates to 99 sources and 1786 estimates), improving the robustness of our analysis.

**Results:**

Results indicated a generally decreasing trend in stunting and a stable, slightly diminishing rate for overweight, with large differences in trends between low- and middle-income countries compared with high-income countries. No differences were found between age groups and between sexes. Cross-validation results indicated that both stunting and overweight models were robust in estimating the indicators for our data (root mean squared error: 0.061 and 0.056; median absolute deviation: 0.045 and 0.042; for stunting and overweight, respectively).

**Conclusions:**

These statistical methods can provide useful and robust information on child malnutrition trends over time, even when data are sparse.

See corresponding editorial on page 1595.

## Introduction

Monitoring countries’ progress toward the achievement of their nutrition targets using national surveys is of great interest in global health. Global rates of childhood stunting prevalence are still unacceptably high and thus a cause for major concern (1, 2); meanwhile, childhood overweight and obesity is increasingly common (3, 4). The 2012 World Health Assembly endorsed a 40% reduction in stunting, and no increases in overweight, for children <5 y of age by 2030 (5). These are also addressed by target 2.2 of the Sustainable Development Goals (SDGs) and the WHO General Program of Work 2019–2023 (GPW13) (6, 7). Monitoring trends in stunting and overweight can be difficult, however, owing to data sparsity, which is a common challenge in nutritional epidemiology and public health. Developments in statistical methods allow us to gain valuable insights using longitudinal data for these indicators, even when data are sparse (8, 9). In this study, we implement these methods to examine the longitudinal trends in stunting and overweight for children under the age of 5 y over a 30-y period—between 1990 and 2020—using existing stunting and overweight data from the WHO European region, where data coverage is poor. We demonstrate a method to produce suitable estimates from sparse data, and therefore a useful tool for monitoring and assessing trends in childhood malnutrition.

Stunting, resulting from chronic malnutrition, indicates past deficient environment and is an accurate marker of inequality (1, 10). Stunting is a useful overall indicator of a child's well-being and a useful marker for future child development, work capacity as an adult, and susceptibility to chronic disease (1, 10). Stunting is also associated with metabolic, physiological, and psychological risk factors for subsequent child overweight and adult overweight (11–13), which are associated with serious health issues such as an increased risk of premature illness and death in adulthood (3, 4). On its own, childhood overweight and obesity is becoming increasingly common (14).

In the WHO European region, data coverage for stunting and overweight in children <5 y old is low. The 2021 edition of the UNICEF-WHO-World Bank Joint Child Malnutrition Estimates (JME)—a comprehensive global database of standardized child malnutrition estimates—recorded only 27 out of the 53 countries in the region as having available data (15). Whereas in other regions several surveys are implemented on a regular basis, most countries in the WHO European region rely heavily on kindergartens to collect data for children <5 y of age. This results in several of the available data sets, from either surveys or studies, covering only a small part of the indicators’ full age range of birth to 5 y. Furthermore, inclusion in the JME database requires the data to cover ≥3 y of the full age interval. These types of data are therefore usually not included in the JME global exercise, even though they are nationally representative and include no major data quality concerns as per the UNICEF and WHO criteria for child anthropometric data (16). Other concerns with sparse data in this region include the sporadic administration of national surveys and a lack of standardized methodology across different data sources. Utilizing all available data is important given the data scarcity, provided appropriate reanalyses are conducted of the raw data whenever available and that adjustments are applied for harmonizing estimates across years and countries. Statistical modeling can be used to accommodate data sparsity by applying trends from data-rich countries and periods to areas and times when data are sparse (8).

This study builds on a previous analysis that used heteroscedastic penalized longitudinal models with multisource summary measures in the WHO African region (9) and its subsequent enhanced version applied at global level for the JME 2021 edition (17), by using additional covariates and modeling features for implementation now in the WHO European region. This study aims to address one of the main concerns in this region by proposing a method to use all available data, even if age intervals covered are shorter than the standard age interval of birth to 5 y, and systematically adjust for differences in age representation (17). Separate models were run for stunting and overweight in children under the age of 5 y. The models used age, sex, and countries’ income classification, which had 3 benefits. First, we used a flexible strategy to leverage data with partial age intervals in situations where complete age intervals were missing. Second, the estimates were stratified on sex to investigate whether any inequalities in malnutrition prevalence existed due to sex, because several studies have found that, for children <5 y old, boys are more likely to be stunted than girls for various reasons (18, 19). Third, World Bank countries’ income classification, using the 2 groups low- and middle-income countries (LMICs) and high-income countries (HICs), was used to adjust for differences between countries’ malnutrition prevalence by income classification and to aid in prediction of country-level trends (2, 11, 20).

Even though stunting is not as pressing an issue in the European region as in other regions (e.g., Africa), low availability of quality data is a major concern in nutritional epidemiology. Target 17.18 of the SDGs calls for increased availability of high-quality data by 2020 (6). We demonstrate that the methods discussed in this article can provide useful information in monitoring health indicators such as child malnutrition from existing sparse longitudinal data.

## Methods

### Data

Child stunting and overweight prevalence for countries within the WHO European region (**Supplemental Text 1**) were compiled from the JME Database (15) and the WHO Global Database on Child Growth and Malnutrition (21, 22). These databases derive childhood malnutrition prevalence estimates from data sources such as national surveys, nationally representative surveys, and representative studies on childhood malnutrition, whereby the recorded prevalence estimates are standardized as per the methods described in the WHO Child Growth Standards (23, 24). These standards provide the global guideline used for monitoring childhood malnutrition; prevalence estimates are standardized using the methods provided in the guideline so that the estimates are comparable across different time periods and different locations. This guideline defines stunting as <2 SDs of height-for-age and overweight as >2 SDs of weight-for-length/height (23). Prevalence estimates with missing sampling standard error (SSE) of the prevalence and missing population size were excluded. Age and sex groups are internal to the data, whereas income classification was created by the authors. Income classification was obtained from the World Bank; countries were determined as LMICs or HICs based on their classification for the last 10 y (25).

A total of 99 and 90 data sources for stunting and overweight, respectively, were available from 26 countries, spanning the years 1990–2020. In this article we use “year” to refer to the calendar year in which a survey was done, distinct from “age” which refers to a child's age, given in months. The data were all collected by October 2020. The data for Greece were not available during the time of this analysis and therefore were not included in our analyses even though the data were included in the 2021 JME database. Including age and sex stratifications, we had a total of 1786 prevalence estimates for stunting and 1769 prevalence estimates for overweight. The prevalence estimates in our data may be stratified by sex and partial age group.

Age is represented as a categorical variable in our data, where the first 5 y of life are split into 6 periods. The 6 periods are 0–5, 6–11, 12–23, 24–35, 26–47, and 48–59 mo, consistent with the age stratification used in standard nutrition surveys. The sex variable has 2 categories of males and females. Countries’ income classification has 2 categories: low-or-middle income or high-income, as classified by the World Bank over the last 10 y (25). **Supplemental Texts 2, 3** and **4** and **Supplemental Tables 1**and**2** further explain the model covariates, detail the data preparation, and provide example data.

Had partial age intervals not been considered, 15 of the 99 stunting (15%) and 10 of the 90 overweight (11%) data sources would have been excluded; in addition, there were only 84 prevalence estimates out of the 1786 (5%) for stunting that spanned the complete age interval (birth to 60 mo) for both sexes (**Table 1**); for overweight, this was only 80 out of 1769 (5%) estimates. By including prevalence estimates with partial age intervals, we have more estimates available for stunting and overweight prevalence than if we only included the complete age interval of birth to 5 y of age. Splitting the complete age estimate into multiple partial age intervals had a small impact on the *effective sample size* compared with adding a new data source, but they helped improve the contribution of predictor variables (26). A total of 28 of the 189 (15%) data sources for both stunting and overweight did not include partial age intervals. There were more prevalence estimates with partial age intervals recorded after the year 2000; this came with an increase in the number of surveys generally after the year 2000.

**TABLE 1:** Summary statistics for stunting and overweight prevalence estimates as rates (1 equaling to 100% of the population), stratified by age and sex, of children under the age of 5 y for countries in the European region between 1990 and 2020^1^

	Stunting			Overweight		
Age group	Both sexes	Girls	Boys	Both sexes	Girls	Boys
0–59 mo						
*n*	84	66	66	80	66	66
Mean ± SD	0.14 ± 0.09	0.14 ± 0.08	0.15 ± 0.09	0.11 ± 0.06	0.11 ± 0.06	0.12 ± 0.07
Min–max	0.01–0.40	0.01–0.38	0.00–0.41	0.03–0.30	0.02–0.31	0.02–0.29
0–5 mo						
*n*	65	63	64	65	63	64
Mean ± SD	0.12 ± 0.07	0.11 ± 0.08	0.13 ± 0.08	0.09 ± 0.05	0.09 ± 0.06	0.10 ± 0.06
Min–max	0.02–0.39	0.01–0.43	0.01–0.41	0.01–0.26	0.00–0.33	0.00–0.26
6–11 mo						
*n*	71	68	68	70	68	68
Mean ± SD	0.11 ± 0.08	0.10 ± 0.08	0.13 ± 0.09	0.11 ± 0.07	0.11 ± 0.07	0.11 ± 0.07
Min–max	0.01–0.39	0.00–0.33	0.01–0.43	0.02–0.28	0.01–0.33	0.02–0.31
12–23 mo						
*n*	72	68	68	71	68	68
Mean ± SD	0.16 ± 0.09	0.15 ± 0.09	0.18 ± 0.10	0.14 ± 0.09	0.13 ± 0.08	0.15 ± 0.09
Min–max	0.01–0.44	0.01–0.41	0.00–0.45	0.03–0.34	0.01–0.39	0.03–0.37
24–35 mo						
*n*	72	69	69	71	69	69
Mean ± SD	0.18 ± 0.12	0.17 ± 0.11	0.19 ± 0.13	0.12 ± 0.07	0.12 ± 0.07	0.13 ± 0.07
Min–max	0.01–0.50	0.00–0.50	0.00–0.51	0.03–0.36	0.01–0.37	0.03–0.36
36–47 mo						
*n*	70	67	67	69	67	67
Mean ± SD	0.15 ± 0.10	0.15 ± 0.10	0.15 ± 0.11	0.11 ± 0.07	0.10 ± 0.07	0.12 ± 0.07
Min–max	0.00–0.44	0.00–0.45	0.00–0.43	0.02–0.28	0.02–0.29	0.01–0.29
48–59 mo						
*n*	71	68	68	70	68	68
Mean ± SD	0.13 ± 0.09	0.13 ± 0.10	0.12 ± 0.10	0.10 ± 0.06	0.09 ± 0.07	0.11 ± 0.07
Min–max	0.00–0.45	0.00–0.46	0.00–0.45	0.01–0.30	0.00–0.28	0.00–0.35
Other partial groups^2^						
*n*	121	110	110	115	109	109
Mean ± SD	0.17 ± 0.10	0.15 ± 0.09	0.17 ± 0.09	0.12 ± 0.07	0.12 ± 0.07	0.13 ± 0.07
Min–max	0.01–0.45	0.01–0.45	0.01–0.46	0.03–0.31	0.03–0.33	0.03–0.32

1 Additional details are provided in the text. Stunting is defined as <2 SDs of height-for-age; overweight as >2 SDs of weight-for-length/height. Our data were compiled from the 2021 Joint Malnutrition Estimates and the WHO Global Database on Child Growth and Malnutrition.

2 Additional details are provided in the text. These are age groups with nonstandard intervals and do not fit into any of the above groups.

Of the 26 countries, only 17 and 18 countries had ≥3 surveys with data over the complete age interval available for stunting and overweight, respectively, for the 30-y period of interest from 1990 to 2020. For the remaining countries, 4 countries had 2 data sources and 5 countries had 1 data source for stunting. For overweight, 3 countries had 2 data sources and 7 countries had 1 data source. One of the objectives of this modeling was to predict prevalence estimates for countries and years where data were missing. For both stunting and overweight, few data sources existed in the period of 1990–1994; the data coverage improved marginally in subsequent years. In the periods 2010–2014 and 2015–2020, the data coverage dropped again. The data set contained an average of 22 and 21 age-group-specific prevalence estimates per country over a 30-y period for stunting and overweight, respectively.

### Statistical analysis

Penalized longitudinal models with heterogeneous error terms were implemented, where the nonlinear longitudinal patterns in the outcomes were captured using penalized cubic B-splines (P-splines). Among-country heterogeneity in the longitudinal pattern was captured using country-specific intercepts and cubic B-splines. The model was fit using the lme function in R (25, 27) and used the connection between P-splines and random-effect models proposed by Currie and Durban (8, 27).

Our model for stunting and overweight prevalence was designed to capture the unique aspects of the data. In total, our model consisted of a linear mixed model with penalized cubic B-splines (P-splines) and a heterogeneous error term on logit-transformed malnutrition prevalence (9, 28). This model has 4 main components. First, the nonlinear longitudinal patterns in the outcomes over time were captured using penalized cubic B-splines (P-splines). Specifically, all models used cubic B-splines spaced 2 y apart over the total study period (1990–2019). Penalizing promotes small B-spline coefficients and a linear pattern. P-splines optimally adapt the penalty to the degree of nonlinearity in the data (8). Second, the SSE values were used to account for increasing residual variance with the SE of the survey. Third, we added to the model the covariates age, sex, and countries’ income classification. Fourth, random intercepts and random B-splines were used to account for among-country heterogeneity. The random B-splines were evenly spaced over the study period; their number and covariance were determined through a model selection process based on the Akaike information criterion with correction (AICc) (29). The model was fit using the statistical software R with the nlme package (25). This method is an extension of previously published methodology for penalized longitudinal models applied to childhood malnutrition and the software program used for this model can be found on GitHub (9, 30). **Supplemental Text 2**provides further details on statistical methodology.

Two surveys had a recorded stunting prevalence of 0, which could not be incorporated due to the logit transformation on the outcome. To circumvent this, we considered these instances to be a limit-of-detection problem, whereby a nonzero prevalence could not be detected. In keeping with the limit-of-detection literature, each such prevalence was replaced with the value 1/2*n*, *n* being the unweighted sample size for the survey (31).

A *k*-fold cross-validation was conducted to check the validity and robustness of our model, where the data were randomly divided into *k* number of groups at the survey level. One group was then taken out and the model rerun on the remaining *k *− 1 groups; this was repeated *k* times. Subsequently, coverage probability of uncertainty intervals, bias, root mean squared error (RMSE), and median absolute deviation (MAD) were calculated for each subset and then averaged across the *k* subsets to assess model performance (32).

## Results

To decide the best covariance structure and number of splines for the model, we checked the AICc statistic for various model configurations. Based on the AICc, a compound symmetry covariance structure for the random effects was chosen for both stunting and overweight models. The penalized splines were equally spaced at every 2 y for the stunting model and at every 4 y for the overweight model. Cross-validation indicated that the selected model was valid and robust. Refer to **Supplemental Text 5** for further details on cross-validation.

**FIGURE 1:**
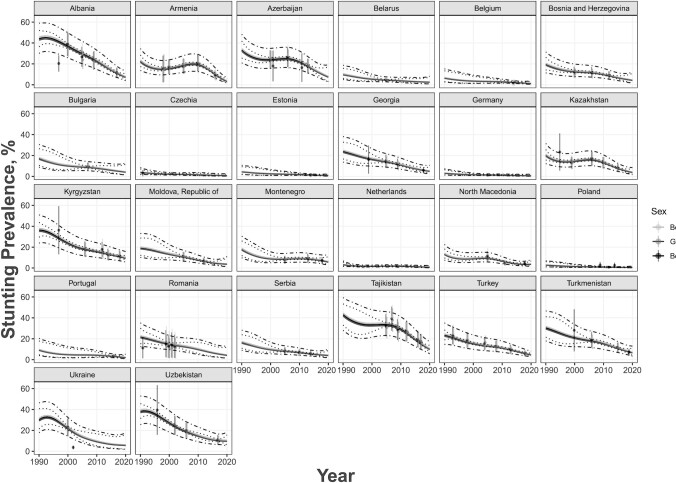
Age group–adjusted and sex-stratified stunting prevalence estimates by country and year for 26 European countries in 1990–2020 for ages 0–59 mo. Sex groups are denoted by different shading as shown in the legend. Estimates for both sexes were from a database with sexes combined. Predicted estimates are denoted by the solid gray line, 95% CIs in dotted gray lines, and prediction intervals in dot-and-dash gray lines.

**FIGURE 2:**
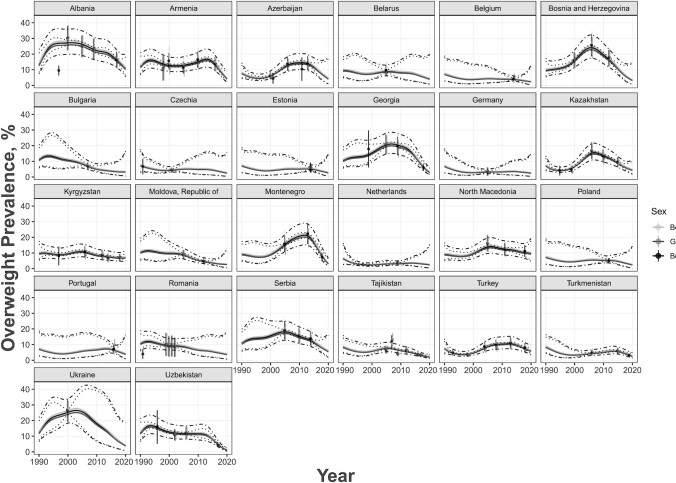
Age group–adjusted and sex-stratified overweight prevalence estimates by country and year for 26 European countries in 1990–2020 for ages 0–59 mo. Sex groups are denoted by different shading as shown in the legend. Estimates for both sexes were from a database with sexes combined. Predicted estimates are denoted by the solid gray line, 95% CIs in dotted gray lines, and prediction intervals in dot-and-dash gray lines.

**FIGURE 3:**
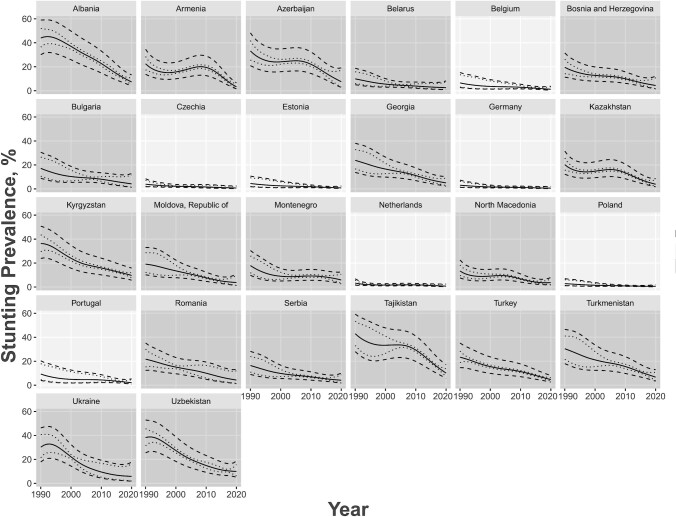
Age group–adjusted and sex-stratified stunting prevalence estimates by country and year for 26 European countries in 1990–2020 for ages 0–59 mo and sexes combined, with the country's World Bank income classification accounted as an additional covariate. Income classification groups are denoted by different shading as shown in the legend. Predicted estimates are denoted by the solid gray line, 95% CIs in dotted gray lines, and prediction intervals in dashed gray lines.

**FIGURE 4:**
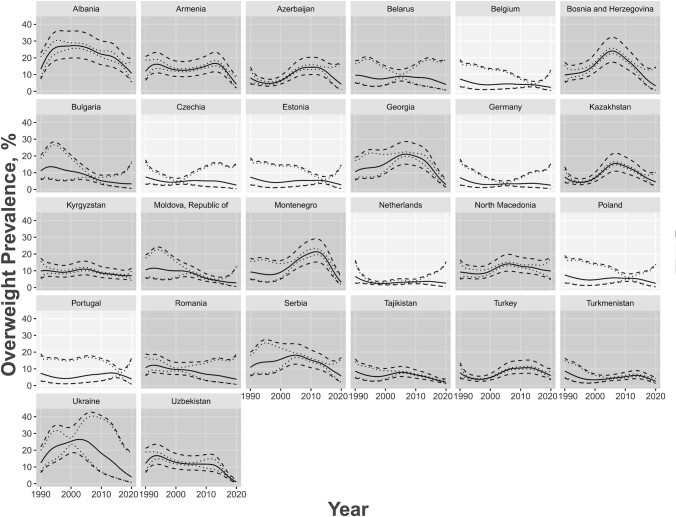
Age group–adjusted and sex-stratified overweight prevalence estimates by country and year for 26 European countries in 1990–2020 for ages 0–59 mo and sexes combined, with the country's World Bank income classification accounted as an additional covariate. Income classification groups are denoted by different shading as shown in the legend. Predicted estimates are denoted by the solid gray line, 95% CIs in dotted gray lines, and prediction intervals in dashed gray lines.

Stunting prevalence had a generally decreasing trend between 1990 and 2020 (**Figure 1**), whereas overweight prevalence had a generally increasing trend which subsequently declined (**Figure 2**). Stunting and overweight prevalence did not differ between age groups or between sexes. Regarding income classification, LMICs tended to have higher rates of stunting with a sharper decline over the 30-y period, whereas HICs had an overall stable rate of stunting consistently close to 0 over the years (**Figure 3**). Similar trends occurred for overweight; HICs had a more stable prevalence of overweight over the years than LMICs (**Figure 4**). For both indicators, there were fewer observed prevalence estimates for HICs than for LMICs, which may have contributed to the stable fitted values over time for HICs. Furthermore, data from LMICs had higher SEs than data from HICs.

Most of the observed prevalence estimates fit well within the range of the predictive intervals. The few estimates that did not fall within this range did not have a recorded SSE associated with the prevalence. Cross-validation results revealed that uncertainty intervals for stunting were appropriate, whereas the overweight intervals were too narrow, which may be due to the elasticity of weight-related indicators. We also observed no important differences in the prediction errors from the *k*-fold cross-validation, even between age groups, for both stunting and overweight. This implied our fitted models were valid and robust for the data observed.

Table 1 presents summary statistics for stunting and overweight prevalence estimates for children <5 y old in the European region from 1990 to 2020, stratified by age and sex. **Table 2** presents summary statistics of these prevalence estimates stratified by age and income. We observed similar trends to the model-derived estimates found in Figures 1–4.

**TABLE 2:** Summary statistics for stunting and overweight prevalence estimates as rates (1 equaling to 100% of the population), stratified by age and income classification, of children under the age of 5 y for countries in the European region between 1990 and 2020^1^

	Stunting		Overweight	
Age group	LMICs	HICs	LMICs	HICs
0–59 mo				
*n*	188	28	186	26
Mean ± SD	0.16 ± 0.08	0.02 ± 0.01	0.12 ± 0.06	0.05 ± 0.02
Min–max	0.04–0.41	0.00–0.03	0.03–0.31	0.02–0.10
0–5 mo				
*n*	177	15	177	15
Mean ± SD	0.13 ± 0.08	0.04 ± 0.02	0.10 ± 0.06	0.03 ± 0.02
Min–max	0.01–0.43	0.01–0.06	0.00–0.33	0.01–0.07
6–11 mo				
*n*	189	18	188	18
Mean ± SD	0.12 ± 0.08	0.02 ± 0.01	0.12 ± 0.07	0.03 ± 0.02
Min–max	0.02–0.43	0.00–0.05	0.01–0.33	0.02–0.08
12–23 mo				
*n*	190	18	189	18
Mean ± SD	0.18 ± 0.09	0.03 ± 0.02	0.15 ± 0.09	0.05 ± 0.02
Min–max	0.04–0.45	0.00–0.11	0.02–0.39	0.01–0.09
24–35 mo				
*n*	189	21	188	21
Mean ± SD	0.20 ± 0.11	0.01 ± 0.01	0.13 ± 0.07	0.07 ± 0.05
Min–max	0.02–0.51	0.00–0.03	0.01–0.37	0.02–0.19
36–47 mo				
*n*	180	24	179	24
Mean ± SD	0.17 ± 0.09	0.01 ± 0.01	0.12 ± 0.07	0.04 ± 0.01
Min–max	0.02–0.45	0.00–0.03	0.01–0.29	0.02–0.06
48–59 mo				
*n*	183	24	182	24
Mean ± SD	0.14 ± 0.09	0.01 ± 0.01	0.10 ± 0.07	0.05 ± 0.05
Min–max	0.01–0.46	0.00–0.05	0.00–0.35	0.00–0.26
Other partial groups^2^				
*n*	334	7	326	7
Mean ± SD	0.17 ± 0.09	0.02 ± 0.01	0.12 ± 0.07	0.04 ± 0.01
Min–max	0.01–0.46	0.01–0.04	0.03–0.33	0.03–0.05

1 Additional details are provided in the text. Stunting is defined as <2 SDs of height-for-age; overweight as >2 SDs of weight-for-length/height. Our data were compiled from the 2021 Joint Malnutrition Estimates and the WHO Global Database on Child Growth and Malnutrition. HICs and LMICs were grouped as per the World Bank Income Classification scheme. HIC, high-income country; LMIC, low- and middle-income country.

2 Additional details are provided in the text. These are age groups with nonstandard intervals and do not fit into any of the above groups.

Our 10-fold cross-validation results indicated that the models were robust in estimating the indicators for our data which included incomplete age intervals. Refer to **Table 3** for the resulting metrics derived from cross-validation and Supplemental Text 5 for a further explanation of these metrics. The coverage probability for stunting was close to 95%, whereas for overweight it was lower at 85.5%, indicating that the SE was underestimated. Bias was close to 0 for both models, and the RMSE values were low: 0.061 for stunting and 0.056 for overweight. The MAD of 0.045 for stunting indicated that our predictions will be within 0.045 of the observed value half the time; the MAD for overweight at 0.042 was similarly close. These results were desirable and indicated that both stunting and overweight models were robust in estimating the indicators for our data which included incomplete age partitions.

**TABLE 3:** Estimates of coverage probability, bias, test errors, and MAD obtained from 10-fold cross-validation for stunting and overweight^1^

	Stunting	Overweight
Coverage probability	0.938	0.855
Average bias	0.006	–0.002
Median bias	0.002	–0.005
Mean squared error	0.004	0.003
RMSE	0.061	0.056
MAD	0.045	0.042

1 MAD, median absolute deviation; RMSE, root mean squared error.

To determine if there were any differences in RMSE and MAD values between age groups, a random-effect ANOVA of the RMSE and MAD on age group was run for both stunting and overweight (**Tables 4** and **5**). The RMSE values were not different between age groups for the stunting and overweight estimates (*P* = 0.782 and *P* = 0.995, respectively); MAD values were not different between age groups either for both stunting and overweight estimates (*P* = 0.139 and *P* = 0.994, respectively).

**TABLE 4:** RMSE and MAD values from cross-validation by age group for stunting^1^

	RMSE		MAD	
Age group, mo	Mean ± SD	95% CI	Mean ± SD	95% CI
0–5	0.0159 ± 0.002	0.0125, 0.0194	0.0125 ± 0.001	0.0099, 0.0151
6–11	0.0149 ± 0.002	0.0115, 0.0184	0.0088 ± 0.001	0.0062, 0.0114
12–23	0.0140 ± 0.002	0.0106, 0.0175	0.0077 ± 0.001	0.0051, 0.0104
24–35	0.0168 ± 0.002	0.0133, 0.0202	0.0114 ± 0.001	0.0088, 0.0140
36–47	0.0141 ± 0.002	0.0106, 0.0175	0.0098 ± 0.001	0.0072, 0.0124
48–59	0.0138 ± 0.002	0.0103, 0.0172	0.0095 ± 0.001	0.0069, 0.0121

1 MAD, median absolute deviation; RMSE, root mean squared error.

**TABLE 5:** RMSE and MAD values from cross-validation by age group for overweight^1^

	RMSE		MAD	
Age group, mo	Mean ± SD	95% CI	Mean ± SD	95% CI
0–5	0.0540 ± 0.002	0.0504, 0.0577	0.0408 ± 0.001	0.0381, 0.0436
6–11	0.0551 ± 0.002	0.0517, 0.0586	0.0419 ± 0.001	0.0393, 0.0445
12–23	0.0541 ± 0.002	0.0508, 0.0574	0.0411 ± 0.001	0.0386, 0.0436
24–35	0.0537 ± 0.002	0.0502, 0.0571	0.0408 ± 0.001	0.0381, 0.0434
36–47	0.0539 ± 0.002	0.0504, 0.0575	0.0412 ± 0.001	0.0385, 0.0439
48–59	0.0540 ± 0.002	0.0506, 0.0575	0.0412 ± 0.001	0.0386, 0.0438

1 MAD, median absolute deviation; RMSE, root mean squared error.

## Discussion

The methods used in this study were primarily intended to accurately track changes in childhood stunting and overweight in the WHO European region, where data were sparse and data with complete age intervals were not always possible to obtain. We developed penalized longitudinal models with multisource summary measures to estimate stunting and overweight prevalence with their uncertainties for all data available that met the inclusion criteria. The model estimates were obtained using data from any country which had ≥1 survey estimate for stunting or overweight. We specified models adjusted for age group using age partition covariates, stratified for sex, and adjusted for the countries’ income group. SSEs were imputed where they were missing in the original data set. Cross-validation was conducted to assess the validity and robustness of the model, accounting for all age intervals in the analysis data set, not only those covering birth to 5 y.

This method has several strengths. First, it is an effective way to produce estimates from sparse data, due to either lack of surveys conducted for a country in a year or the different sampling frames for age intervals. Second, the method is specifically designed to account for the unique aspects in the data, mainly modeling nonlinear trends in prevalence over time with partial age intervals and incorporating missing and observed SSEs and among-country heterogeneity. Third, this method is useful to compare data collected using standardized procedures with those collected from alternative data sources where procedures are not necessarily standardized (e.g., pediatric clinics) and to subsequently evaluate the impact of data quality on prevalence estimates. Fourth, code, sample data, and examples for how to implement the model are publicly available (9, 30). Future research is needed to examine how the landscape looks depending upon which data source is used.

A challenge when using this method is the mathematical complexity of this model. Despite the straightforward implementation programmatically, some understanding of statistical methods is required to appropriately fit mixed-effects models and to select the appropriate model parameters, such as covariance structure and number of splines. Another is that prediction errors could be difficult to use when judging model fit. Prediction errors for this model were heavily driven by the overall prevalence estimate; errors will be smaller if a prevalence estimate is closer to 0 than if it is closer to 0.5. Another potential challenge for using this method is in determining whether one has enough observed prevalence estimates to conduct the modeling process. Even though the objective of this method is to assist in predicting prevalence where there is data sparsity, a minimal frequency of observed prevalence estimates is needed to derive results that are accurate and informative in monitoring countries’ progress. Cross-validation must therefore be done postestimation to ensure the fitted model is valid and robust.

In conclusion, monitoring childhood malnutrition prevalence and trends, especially as manifested by stunting and overweight, is a global health public concern. Deriving useful information on the trends of childhood stunting or overweight from sparse longitudinal data is a useful exercise in line with target 17.18 of the SDGs for improved data quality. These methods can be repeated for other regions that aim to monitor trends in their countries’ levels of childhood malnutrition despite sparse data. Assessing these trends can provide important information to policy makers as they examine the effectiveness of nutrition programs over time or identify priority areas for action.

Our method accounted for age partition, sex, and income classification to estimate differences in stunting and overweight prevalence in the WHO European region. The trends in stunting and overweight prevalence differed between LMICs and HICs, justifying the proposed adjustment by income group and increasing the estimates’ accuracy. Although prevalence did not differ between age groups and between sexes for these indicators in this region, sex-stratified reporting and monitoring is important to allow for the expected inequality analysis, emphasized in the SDGs. The cross-validation showed no difference in the prediction errors between age partitions. Data from LMICs had higher SEs than data from HICs, which is mainly due to the prevalence estimates for LMICs being closer to 0.5 (where uncertainty is maximized) but perhaps also due to variability in the LMICs’ nutritional status. Nevertheless, validation techniques indicated the models were largely accurate and unbiased for both stunting and overweight.

Although aiming to fill in data gaps in the European region, this analysis reiterates the importance of both collecting anthropometric data across the entire birth-to-5 y age interval and improving practices that enhance data quality (16). Even with data sparsity, carefully developed and applied statistical methods such as penalized longitudinal models allowed us to generate robust estimates of trends in childhood malnutrition indicators for areas with sparse data.

## Supplementary Material

nxac072_Supplemental_File

## Data Availability

The complete data used for the analysis were obtained from the UNICEF-WHO-World Bank Joint Child Malnutrition Estimates (JME) 2021 edition and the WHO Global Database on Child Growth and Malnutrition. The data set on the JME website can be downloaded under the heading “Download” via the subheading “Joint data set including survey estimates,” which links directly to an Excel spreadsheet.

## Notes

Supported by the WHO and the European Commission through the Science and Technology in childhood Obesity Policy (STOP) project.

Author disclosures: the authors report no conflicts of interest.

Supplemental Texts 1–5 and Supplemental Tables 1 and 2 are available from the “Supplementary data” link in the online posting of the article and from the same link in the online table of contents at https://academic.oup.com/jn/.

Abbreviations used: AICc, Akaike information criterion with correction; HIC, high-income country; JME, UNICEF-WHO-World Bank Joint Child Malnutrition Estimates; LMIC, low- and middle-income country; MAD, median absolute deviation; RMSE, root mean squared error; SDG, Sustainable Development Goal; SSE, sampling standard error.
