# Supplementary material for: Estimating Childhood Stunting and Overweight Trends in the European Region from Sparse Longitudinal Data
Source: J Nutr. 2022 Mar 29;152(7):1773–82. doi: 10.1093/jn/nxac072 (PMC9258559; doi:10.1093/jn/nxac072)
Supplement: nxac072_Supplemental_File [file nxac072_supplemental_file.docx]

# Estimating childhood stunting and overweight trends in the European region from sparse longitudinal data. Chitra M. Saraswati. Online Supplementary Material

# Supplemental Text 1. WHO European Region

The WHO European region include the following countries: Albania, Andorra, Armenia, Austria, Azerbaijan, Belarus, Belgium, Bosnia and Herzegovina, Bulgaria, Croatia, Cyprus, Czechia, Denmark, Estonia, Finland, France, Georgia, Germany, Greece, Hungary, Iceland, Ireland, Israel, Italy, Kazakhstan, Kyrgyzstan, Latvia, Lithuania, Luxembourg, Malta, Monaco, Montenegro, Netherlands, North Macedonia, Norway, Poland, Portugal, Republic of Moldova, Romania, Russian Federation, San Marino, Serbia, Slovakia, Slovenia, Spain, Sweden, Switzerland, Tajikistan, Turkey, Turkmenistan, Ukraine, United Kingdom of Great Britain and Northern Ireland, Uzbekistan.

# Supplemental Text 2. Model specification

For both stunting and overweight, we implemented a penalised longitudinal model with heterogenous error terms, where the non-linear longitudinal patterns in prevalence over time were captured using penalised cubic B-splines. The knots of the penalised cubic B-splines were chosen to be dense (every 2 years) and penalisation is used to avoid overfitting the data. The penalty term is estimated from the model using the connection between penalised B-splines and random effect models (1). Between-country heterogeneity in the longitudinal pattern is captured using country-specific random intercepts and random cubic B-splines. The random B-splines were evenly spaced, the number and covariance of the random B-splines was chosen using AICc. More specifically, we tested 2 or 3 random B-splines with 3 different covariance matrices (unstructured, exchangeable, and independent). As a result, there were 6 random B-splines models tested for each outcome. The analysis was run using the most recent program by Dr. Alexander McLain based on his 2019 publication (2). A version available to the public can be found on GitHub (3). The following section provides a summary of the model specification as outlined in the 2019 article by McLain et al.

Let $D_{i}=\left\{ \mathbf{Y}_{i}, \mathbf{X}_{i}, \mathbf{t}_{i}, \mathbf{S}_{i} \right\}$ denote the observed data for country $i$ whereby $\mathbf{Y}_{i}$, $\mathbf{X}_{i}$, $\mathbf{t}_{i}$, and $\mathbf{S}_{i}$ are $n_{i}$ vectors of the outcome, covariates, time, and heterogeneity data respectively. The unpenalised log-likelihood for the $i$-th country, denoted by $l_{i}(\boldsymbol{\theta}|D_{i})$, is formed by noting that $\mathbf{Y}_{i}$ is multivariate Gaussian with mean $\beta^{*}'X_{i}^{*}+\gamma'\mathbf{B}_{i}$ and covariance matrix $\mathbf{B}_{i}^{r}\sigma_{b}\mathbf{B}_{i}^{r}'+\sigma^{2}\mathbf{V}_{i}(\mathbf{S}_{i};\delta)$, where $\mathbf{B}_{i}=\left[ \mathbf{B}_{ij} \right]$ and $\mathbf{B}_{i}^{r}=[\mathbf{B}_{ij}^{r}]j=1,\ldots, n_{i}$ are column-stacked matrices of spline functions with dimensions of $K\times n_{i}$ and $K^{r}\times n_{i},$ respectively. Here, $\mathbf{V}_{i}\left( \mathbf{S}_{i};\delta\right)=\delta+\mathbf{S}_{i}^{\mathbf{2}}$ accounts for the increasing residual variance with the SSE. Specifically, the $\delta$ term accounts for the amount of sampling and non-sampling error in the data. For example, if $\delta=0$ then all the error in the model is due to the sampling error, while if $\delta_{1}$ is large most of the error is due to non-sampling error.

We assume the penalisation can be stated through a $2nd$-order difference in the B-spline coefficients. Assuming $N$ independent replicates of the data $D=D_{1},\ldots,D_{N}$ are observed, the penalised likelihood is:

$l^{p}(\boldsymbol{\theta}|D)=\sum_{i=1}^{N} l_{i}\left( \boldsymbol{\theta}|D_{i} \right)-\lambda\gamma'\mathbf{P}_{2}'\mathbf{P}_{2}\gamma$,

where $\mathbf{P}_{2}$ is a matrix which induces the second order differences of $\gamma$, and $\lambda$ is a penalty term used to control the smoothness of the curves. The penalised likelihood above can be optimised via its equivalence with a mixed-model (1). The penalised model is estimated using the lme function in R’s nlme package (4). Code and sample data to fit the model can be downloaded from GitHub (3).

**Missing sampling standard errors**

The surveys have varying sample sizes and sampling strategies; the prevalence estimates’ sampling standard errors (SSE) are therefore important in quantifying precision. However, we found that many of the surveys in our database report prevalence estimates without SSE values. A reliable method is therefore needed to incorporate them. To do this, surveys with complete information were used to estimate a model for the SSE values; the model used to predict SSE was a log-regression function of the observed prevalence, sample size, and survey type. The type of survey is included to account for the different sampling strategies employed by different organisations such as the DHS, MICS, and others which include nationally representative data obtained from studies, which is explained further in the main text. Specifically, the model takes the form

$log(SSE)=\beta_{0}+\beta_{1}\log\left[ Y_{ij}\left( 1-Y_{ij} \right) \right]+\beta_{2}\log{(n}_{ij})+\beta_{3}\mathrm{Type}_{ij}+d_{i}+\varepsilon_{ij}$,

where $Y_{ij}$ is the prevalence estimate for country $i$ in year $j$, $n_{ij}$ is the sample size for country $i$ in year $j$, Type is the survey type, and $d_{i}$ is a country-specific random intercept. Single imputation of the missing SSE values is then used (McLain et al., 2019, found single and multiple imputation worked similarly). The imputed SSE is given by:

$$\overset{̂}{SSE}=e^{\mu_{ij}+\sigma_{ij}}$$

whereby $\mu_{ij}$ is the predicted value of the $\log(SSE)$ according to the log-regression function given above, and the standard error $(\sigma_{ij})$ is added to increase the uncertainty for those sources that require SSE imputation. This log regression function was run using the lme function in R’s nlme package.(4)

# Supplemental Text 3. Model covariates

**Partial age intervals**

As mentioned, the data points obtained can be so sparse that sometimes, only partial age intervals are captured. To overcome this, data with partial age intervals are still incorporated into the modelling process as an additional covariate. The data points with partial age intervals are still utilised to estimate the malnutrition prevalence for the whole age interval. This is done through ‘partitioning’ the partial age intervals. Partitioning the partial age intervals, or the age sub-groups, involve breaking the whole age interval of 0-60 months to six sub-groups or ‘partitions’. The following sub-division is used:

- Partition 1 (P1) includes the age interval of 0-6 months;
- P2 includes the age interval of 6-12 months;
- P3 includes the age interval of 12-24 months;
- P4 includes the age interval of 24-36 months;
- P5 includes the age interval of 36-48 months; and
- P6 includes the age interval of 48-60 months.

These partitions are dummy variables that reflects the age interval captured in the data. As an example, a data point covering the whole age interval of 0-60 months would be denoted as ‘1’ for all variables P1 through to P6. A data point covering the age interval 2-5 years, i.e. 24-60 months, would be denotes as ‘0’ for variables P1, P2 and P3 and ‘1’ for variables P4, P5 and P6. In the instance of non-standard age intervals, the overlapping partition is still included; i.e. in the instance of 0.25 to 5 years, this data point will be denoted ‘1’ for all variables P1 through to P6.

An example of what this set of covariates would look like is included in Supplementary Table 2. We remove the first partition P1 from our list of covariates as it would otherwise introduce the issue of perfect collinearity.

**Sex grouping**

For the final model, in addition to age adjustment and country income, the model was expanded to include sex as a factor. This allowed for the generation of stunting and overweight estimates stratified by sex. The following covariates were included:

- BS is a binary variable which was coded ‘1’ if the observation included both sexes;
- SM is a binary variable which was coded ‘1’ if the observation is for males only; and
- SF is a binary variable which was coded ‘1’ if the observation is for females only.

# Supplemental Text 4. Data preparation

Both models require a specific format for the data input. A major component of this exercise, therefore, is preparation of the data to comply with the format required for modelling. Specifically, the following steps were conducted to prepare the data for analysis. These steps were done separately for the stunting and overweight data.

1. Subset the observations of interest. In this exercise, we are interested in: a) a non-missing value for the malnutrition indicator of interest; b) a non-missing non-weighted sample size to impute missing SSEs; and c) data at the national level, i.e. not stratified for sex, area, geographical region, wealth quintile, or mother’s education. We also remove data points where the sample covers age intervals beyond 0-5 years.
2. Subset the variables of interest. In this exercise, data is subset for each malnutrition indicator we want to run the analysis on (i.e. stunting or overweight), sex, and age group only.
3. Transform the stunting and overweight prevalence to proportions (i.e. transforming the prevalence range from 0 to 100 to that of a proportion ranging from 0 to 1) and their corresponding standard errors.
4. Generate age group partitions, i.e. the variables P1 to P6 as previously discussed in Appendix 2. This was done through hard coding based on the age groups that are in the existing dataset.
5. Generate dummy variables for sex, i.e. the variables ‘BS’, ‘SM’ and ‘SF’ as previously discussed in Appendix 2.
6. Create rows for each country by year by age group and by sex combination that we would like the model to predict estimates for. We ultimately have at least 19,530 data points with standard age groupings; some data points may fall outside of the standard age interval, leading to more data points. This number is obtained from a combination of 31 countries, 30 years, seven age groupings, and three sex groupings ($31\times30\times7\times3=19,530$).
7. Impute missing SSE, as outlined in the section on Modelling. SSE values were generated in 3 instances for stunting and in no instances for overweight.
8. Add the countries’ income classification as an additional covariate in our model. This classification specifies whether a country is considered low-or-middle income or high-income, as specified by the World Bank, consistently over the last ten years.

The program specifically requires one row in the input data for each country-year-covariate combination the user wants to generate predictions for. Refer to Supplemental Table 1 for an example of the main input for analysis and Supplemental Table 2 for an example of the input of covariates. In our instance, there are six main variables (country, year, prevalence estimate, SE of estimate, estimated sampling standard error, and SE of SSE) and eight covariates (P2, P3, P4, P5, P6, SM, SF and WB).

# Supplemental Text 5. Cross-validation

A 10-fold cross-validation was implemented in this exercise.(5) First, the data points were grouped at the survey level; that is, data points from the same source, even with the differing age groups, are considered as one ‘survey’. This set of surveys is then randomly split into ten non-overlapping groups, or ‘folds’. Each of these tenths are considered the ‘validation set’ and the remainder are the ‘training set’.

Starting with the first validation set, we take out the validation set and fit our model on the remaining training set. We then compute the following based on data points in the training set: coverage probability, average bias, median bias, mean squared error (MSE), root MSE, and median absolute deviation. We repeat this procedure ten times where each time, a different validation set is used. We therefore obtain ten estimates; the 10-fold cross-validation estimate is computed by averaging these values.

Coverage probability is the proportion of times the 95% prediction interval of the estimated summary mean contains the true value. It is desirable to have a coverage of near 95%. Bias is the average difference between the true (simulated) mean and its estimate across the 10 simulation replicates; it is desirable to have a bias near zero. The mean squared error (MSE) is the average squared difference between the true (simulated) mean and its estimate across the ten simulation replicates, and it is desirable to have an MSE close to zero. The root mean squared error (RMSE) can be interpreted as the standard deviation of the unexplained variance, which indicates the distribution of our errors. It is in the same unit as our response variable and lower values of RMSE indicate a better fit of the model. Finally, the median absolute deviation (MAD) provides a robust measure for the spread of our data.

**Supplemental Table 1.** An example of the main input for analysis.

| **Country** | **Year** | **Y**^1^ | **SE_var** | **SE_pred** | **SE_pred_SE** |
| --- | --- | --- | --- | --- | --- |
| ALB | 2000 | 0.3915548 | 0.0215892 | 0.01968386 | NA |
| ALB | 2000 | 0.3874635 | 0.0599717 | 0.05896529 | NA |
| ALB | 2000 | 0.3944845 | 0.0589265 | 0.06136705 | NA |
| ALB | 2000 | 0.3739524 | 0.0388744 | 0.0407101 | NA |
| ALB | 2000 | 0.5035591 | 0.0366909 | 0.04106956 | NA |
| ALB | 2000 | 0.3433378 | 0.0359457 | 0.03575273 | NA |
| ALB | 2000 | 0.3542901 | 0.041592 | 0.04445121 | NA |
| ALB | 2000 | 0.4014087 | 0.0277085 | 0.02706451 | NA |
| ALB | 2000 | 0.3176965 | 0.0975914 | 0.08250077 | NA |
| ALB | 2000 | 0.4253965 | 0.0730929 | 0.07746109 | NA |
| ALB | 2000 | 0.363217 | 0.0549758 | 0.05529695 | NA |
| ALB | 2000 | 0.5074915 | 0.055553 | 0.05670418 | NA |
| ALB | 2000 | 0.365635 | 0.0443582 | 0.049137 | NA |
| ALB | 2000 | 0.3993222 | 0.0648556 | 0.06398366 | NA |
| ALB | 2000 | 0.3817849 | 0.0261786 | 0.0269806 | NA |
| ALB | 2000 | 0.4349435 | 0.0739678 | 0.077702 | NA |
| ALB | 2000 | 0.334749 | 0.0860914 | 0.09162929 | NA |
| ALB | 2000 | 0.3854382 | 0.0546375 | 0.05649914 | NA |
| ALB | 2000 | 0.4997782 | 0.051578 | 0.05608469 | NA |
| ALB | 2000 | 0.3214488 | 0.0454076 | 0.04894323 | NA |

^1^ The variables $Y$, $SE\_var$ and $SE\_pred$ refer to the malnutrition prevalence of interest (either stunting or overweight), the associated SSE, and the predicted SSE where the SSE is not available respectively.

**Supplemental Table 2.** An example of the input of covariates for analysis, corresponding to the main input.

| **P21** | **P3** | **P4** | **P5** | **P6** | **SM** | **SF** | **WB** |
| --- | --- | --- | --- | --- | --- | --- | --- |
| 1 | 1 | 1 | 1 | 1 | 1 | 1 | 1 |
| 0 | 0 | 0 | 0 | 0 | 1 | 1 | 1 |
| 1 | 0 | 0 | 0 | 0 | 1 | 1 | 1 |
| 0 | 1 | 0 | 0 | 0 | 1 | 1 | 1 |
| 0 | 0 | 1 | 0 | 0 | 1 | 1 | 1 |
| 0 | 0 | 0 | 1 | 0 | 1 | 1 | 1 |
| 0 | 0 | 0 | 0 | 1 | 1 | 1 | 1 |
| 1 | 1 | 1 | 1 | 1 | 1 | 0 | 1 |
| 0 | 0 | 0 | 0 | 0 | 1 | 0 | 1 |
| 1 | 0 | 0 | 0 | 0 | 1 | 0 | 1 |
| 0 | 1 | 0 | 0 | 0 | 1 | 0 | 1 |
| 0 | 0 | 1 | 0 | 0 | 1 | 0 | 1 |
| 0 | 0 | 0 | 1 | 0 | 1 | 0 | 1 |
| 0 | 0 | 0 | 0 | 1 | 1 | 0 | 1 |
| 1 | 1 | 1 | 1 | 1 | 0 | 1 | 1 |
| 0 | 0 | 0 | 0 | 0 | 0 | 1 | 1 |
| 1 | 0 | 0 | 0 | 0 | 0 | 1 | 1 |
| 0 | 1 | 0 | 0 | 0 | 0 | 1 | 1 |
| 0 | 0 | 1 | 0 | 0 | 0 | 1 | 1 |
| 0 | 0 | 0 | 1 | 0 | 0 | 1 | 1 |

^1^ The variables $P2$ to $P6$ are the age partitions mentioned in Supplemental Text 4. The variable $P1$ was removed to prevent the issue of perfect collinearity; we consider it as a reference variable. The variables $SM$ and $SF$ are the dummy sex variables. $WB$is an indicator variable indicating whether the country is classified as low- or middle-income or high income.

# Supplemental References

1. Currie ID, Durban M. Flexible smoothing with P-splines: a unified approach. Statistical Modelling SAGE Publications India; 2002;2:333–49.

2. McLain AC, Frongillo EA, Feng J, Borghi E. Prediction intervals for penalized longitudinal models with multisource summary measures: An application to childhood malnutrition. Statistics in Medicine 2019;38:1002–12.

3. McLain AC. Fitting Penalized Heterogenous Mixture Models [Internet]. North Carolina, USA; 2020 [cited 2022 Feb 24]. Available from: https://github.com/alexmclain/PHMM

4. Pinheiro J, Bates D, DebRoy S, Sarkar D, R Core Team. nlme: Linear and Nonlinear Mixed Effects Models [Internet]. 2021. Available from: https://CRAN.R-project.org/package=nlme

5. James G, Witten D, Hastie T, Tibshirani R. Resampling Methods: Cross-Validation. In: James G, Witten D, Hastie T, Tibshirani R, editors. An Introduction to Statistical Learning: with Applications in R. [Internet] New York, NY: Springer; 2013 [cited 2021 Jul 19]. Available from: https://doi.org/10.1007/978-1-4614-7138-7_3
